# Healthcare professionals’ perspectives on implementing the Swedish palliative care guide in geriatrics – a qualitative study using small-group and individual interviews

**DOI:** 10.1186/s12877-025-06516-1

**Published:** 2025-11-04

**Authors:** Marie-Louise Möllerberg, Karin Dalhammar, Birgit H. Rasmussen, Carl Johan Fürst, Maria E. C. Schelin, Christel Hedman

**Affiliations:** 1 Faculty of Health and Society, Department of Care Science, Malmö University, Malmö, Sweden; 2 Institute for Palliative Care, Lund University and Region Skåne, Lund, Sweden; 3 Faculty of Medicine, Department of Health Sciences, Lund University, Lund, Sweden; 4 Department of Clinical Sciences Lund, Lund University, Lund, Sweden; 5 Department of Molecular Medicine and Surgery, Karolinska Institutet, Stockholm, Sweden; 6 Stockholms Sjukhem Foundation, R&D unit, Stockholm, Sweden

**Keywords:** Clinical pathway, Clinical practice guidelines, Decision support, Early identification, Early palliative care, Group interviews, Health care professionals’ perspective, Palliative care, Person-centered care, Qualitative methods

## Abstract

**Background:**

As the global population ages, the demand for palliative care is expected to increase. However, studies show that older patients often receive inadequate palliative care, partly due to healthcare professionals lacking sufficient training and tools to identify and address palliative care needs effectively. This study aims to explore healthcare professionals’ experiences regarding facilitators and barriers to integrating a palliative care approach in a geriatric setting by implementing the Swedish Palliative Care Guide.

**Methods:**

This qualitative study employed a design consisting of four small-group interviews (with three participants each) and one individual interview, involving a total of 13 healthcare professionals working in a geriatric setting. The aim was to explore their experiences with integrating the Swedish Palliative Care Guide. Data were analyzed using the rapid identification of themes method and inductive content analysis.

**Results:**

The analysis revealed four interrelated themes—organizational prerequisites, knowledge of palliative care, teamwork, and communication—that acted as both facilitators and barriers to integrating the Swedish Palliative Care Guide in a geriatric setting. While structured tools and shared goals supported implementation, challenges such as unclear responsibilities, knowledge gaps, and discomfort discussing end-of-life issues hindered consistent application. Participants emphasized that continuity in care and a shared understanding among team members enhanced the use of the guide. In contrast, short-term admissions and a lack of systemic integration of the Swedish Palliative Care Guide into routine practice pose significant challenges to its long-term sustainability.

**Conclusions:**

Successful implementation of the Swedish Palliative Care Guide in geriatric settings requires clear professional responsibilities, integration into existing digital documentation systems, and targeted training to support staff in initiating end-of-life conversations. Tailoring implementation strategies for local conditions, such as short-term admissions and high staff turnover, can enhance sustainability. These findings provide practical guidance for improving the integration of palliative care approaches into everyday geriatric practice.

**Supplementary Information:**

The online version contains supplementary material available at 10.1186/s12877-025-06516-1.

## Background

A palliative care approach should be integrated into the regular care for all patients with palliative care needs, regardless of the diagnosis, care setting, or phase of illness [1, 2]. Despite extensive support needs, studies show a lack of access to palliative care for older adults with multiple illnesses, both from a national [3, 4] and an international perspective [5, 6]. In the final stage of life, older individuals are less likely to receive pain assessments, information about the transition to end-of-life care, pro re nata drugs for common symptoms, and expert consultations for unrelieved pain compared to younger patients [4, 7]. Research also indicates that the general competence in identifying and assessing palliative care needs among nurses working in geriatric hospitals is inadequate [8, 9].

The need for palliative care is expected to increase rapidly as the global population ages. By 2070, the proportion of people aged 70 and older in Sweden is expected to reach one in five, up from one in six in 2022 [10]. This demographic shift presents us with an urgent need for innovative approaches to healthcare, particularly in the two overlapping fields of geriatrics and palliative care [11, 12]. The EAPC White Paper [2] emphasizes the importance of integrating palliative, geriatric, and rehabilitative approaches to meet the complex needs of older adults living with frailty. Early integration of palliative care close to diagnosing a life-threatening illness has demonstrated multiple benefits. For patients, it reduces symptom burden, enhances quality of life, and may prolong survival, particularly in oncology settings [13, 14]. Additionally, palliative care is associated with reduced healthcare utilization [15], including hospital referrals and emergency visits [16]. These reductions not only ease the burden on patients, by avoiding unnecessary hospitalizations, but also allow healthcare systems to reallocate resources to other patients in need. However, among patients with non-cancer illnesses such as chronic obstructive pulmonary disease [17] and heart failure [18], the results are more mixed, showing only modest improvements in symptom burden and no significant enhancement in quality of life [19]. This may reflect the complexity and variability of disease trajectories in non-malignant conditions, where prognostication is more difficult and palliative care needs are less clearly defined. These findings underscore the importance of individualized palliative care approaches, tailored to the specific needs, preferences, and disease progression of each patient [2]. In line with this, a structured end-of-life program that actively involves family members has also been shown to improve patient comfort and satisfaction during the final days of life, as reported by both families and healthcare professionals [20–22].

Integrating a palliative care approach into geriatrics, i.e. Geriatric Palliative Care, could decrease unnecessary suffering for older adults with chronic multimorbidity and functional dependency [2, 23–25]. Both geriatrics and palliative care are based on multiprofessional and interdisciplinary fields of medicine, emphasizing person- and family-centered care to improve quality of life. Integration of palliative care into geriatrics has been shown to enhance symptom control, communication, and has facilitated personalized care tailored to the complex needs of older adults with cancer [26]. Castelo-Loureiro et al. [27] claims that geriatric palliative care should be established as a fundamental element of the healthcare system to enhance the quality of care for this vulnerable and expanding population.

To support healthcare professionals in identifying and addressing palliative care needs, the Swedish Palliative Care Guide (S-PCG) was developed and tested from 2013 to 2016. The S-PCG aims to ensure person-centred care and evidence-based practice throughout the patient’s disease trajectory and meet palliative care needs for all patients, regardless of diagnosis or care setting [28]. This aligns with the EAPC White Paper’s call for structured, interdisciplinary frameworks to guide care for older adults with frailty [2]. The S-PCG is a structured clinical tool (Table 1) designed to support healthcare professionals in delivering person-centred palliative care across different stages of the end-of-life trajectory.

**Table 1:** Description of the S-PCG Intervention. Based on the template for intervention description and replication (TIDieR) framework [29]

TIDieR Element	Description of the S-PCG Intervention
What	The S-PCG consists of three main parts: - Part 1: Assessment of palliative care needs - Part 2: Initiating palliative care (including Part 2D for end-of-life care) - Part 3: Care after death Each part includes structured guidance for assessing physical, psychological, social, and spiritual needs, and supports documentation and care planning.
Why	To improve the quality and consistency of palliative care by providing a structured framework that supports person-centred care, timely symptom management, and shared decision-making.
For whom	Older adults and patients with progressive, life-limiting illnesses, including those with frailty, cancer, or chronic conditions such as COPD and heart failure.
By whom	Healthcare professionals working in geriatric and palliative care settings, including nurses, physicians, physiotherapists, occupational therapists, and social workers.
How	Through structured assessments, documentation templates, and care planning tools embedded in the S-PCG. The guide is used during clinical encounters and team meetings to support decision-making, communication and symptom management.
When	Throughout the last years of life, starting from the identification of palliative care needs (Part 1), through active palliative care and end-of-life care (Part 2 and 2D), and continuing after death with bereavement support and documentation (Part 3).

It offers a comprehensive assessment of physical, psychological, social, and spiritual needs. Thus, it also acts as a decision support system to help healthcare professionals make informed care interventions and encourage care plans tailored to each patient’s needs and preferences [28].

Implementing S-PCG part 2, which supports identifying the patients’ palliative care needs and planning of care in geriatric care, has demonstrated increased patient satisfaction, including communication with healthcare professionals. The authors conclude that the S-PCG has the potential to facilitate the integration of a palliative care approach into hospital-based geriatric care [30]. Despite its use in over 600 healthcare units in Sweden, mainly in older adults’ care, there are no studies on healthcare professionals’ experiences using S-PCG. Although the Care guide is widely implemented, little is known about how it is perceived in practice, what challenges professionals face, and whether it supports the intended goals of person-centred palliative care. This study addresses this gap by exploring healthcare professionals’ experiences regarding facilitators and barriers to integrating a palliative care approach in a geriatric setting through the implementation of the S-PCG. The study also contributes to understanding the extent to which the implementation was successful, in terms of reach, adoption, and perceived usefulness, as part of a broader process evaluation.

## Methods

### Design

This study was part of a larger single-centre study designed to implement S-PCG in a geriatric setting [30]. A qualitative design was employed, incorporating both small-group interviews and an individual interview. While the original aim was to conduct focus group interviews, scheduling constraints and participant availability resulted in smaller groups (comprising three participants), which limited group interaction. Therefore, these are referred to as small-group interviews. One participant was only available for an individual interview, which was conducted using the same semi-structured guide to ensure consistency. This mixed-methods interview approach enabled both collective reflection and individual perspectives, thereby enriching the data.

### Participants

All healthcare professionals who contributed to implementing the use of the S-PCG for the larger study during the implementation and evaluation [30] were eligible participants. A total of 20 individuals met the inclusion criteria and were therefore invited to participate in interviews using purposive sampling (see Table 2 for an overview of participants per interview session).

**Table 2:** Overview of participants for each interview session

Interview Type	Number of Participants	Gender (F/M)	Professional Roles Represented	Age Range	Years of Experience
Group 1	3	3/0	Nurses	29–44	9 months–14 years
Group 2	3	2/1	Physicians, Nurse	36–55	4–28 years
Group 3	3	2/1	Nurse	30–37	3–5 years
Group 4	3	3/0	Nurses	29–36	1–5 years
Individual Interview	1	1/0	Nurse	29	1.5 years
Total	13	11/2	Nurses (*n* = 11), Physicians (*n* = 2)	29–55	9 months–28 years

### Context

The implementation of S-PCG took place in a hospital with one of the largest geriatric clinics in Sweden. The clinic consisted of one ward with 42 beds distributed across three floors, effectively functioning as three separate units. Each floor typically had one senior physician responsible, and the ward was organized into four interprofessional teams. A registered nurse led each team with primary responsibility, while other team members, such as assistant nurses, physiotherapists, and occupational therapists, could be part of multiple teams.

The implementation consisted of two phases. From September to December 2019, the first phase involved introducing S-PCG, including its background, objectives, practical use, education about palliative care specific to geriatric patients, symptom assessment and communication. Structured documentation procedures were adopted before starting the implementation. The routine set-up included collaborative assessments where nurses identified symptoms and explored patient priorities, physiotherapists and occupational therapists evaluated daily functioning, and physicians contributed with medical expertise. Decisions were made in dialogue with the patient and within the interprofessional team, aiming to support person-centred care by integrating the patient’s values, needs, and preferences into the care planning process. The second phase, from January to December 2020, focused on the clinical application of S-PCG. However, this phase was not implemented in patients with cognitive impairment, as the method used in the implementation study was to collect data through patient questionnaires. For a comprehensive overview of the implementation process, see [30].

### Data collection

Of the 20 healthcare professionals who were invited to participate, 13 agreed to take part in the study. They were recruited in February 2021 and interviewed in four small-group and one individual interviews. All interviews were conducted by a senior researcher (CH), experienced in qualitative methods and palliative care. A semi-structured interview guide was used for both small-group and individual interviews. The guide included open-ended questions and follow-up questions about experiences with the S-PCG, perceived facilitators and barriers, and suggestions for improvement. The guide is available as supplementary material (interview guide). All participation was voluntary, and participants were informed that they could withdraw at any time without consequences. Written and oral information about the study was provided, and written informed consent was obtained from all participants prior to both small-group and individual interviews. The interviews were conducted in Swedish, lasted a median of 37 min each (range: 23–42 min), and were audio recorded. The study was approved by the Swedish Ethical Review Authority (DNR 2019–01689).

### Data analysis

Data were analyzed using a rapid identification of themes from audio recordings (RITA) method [31], and qualitative content analysis with an inductive approach [32] was used to identify themes relevant to this study’s aim. Although RITA is often used in time-sensitive evaluations, we selected this method for its structured and transparent approach to theme identification directly from audio material. This was particularly useful given the small size of the interview groups and the need to preserve contextual nuances. The method allowed for efficient and consistent coding while maintaining analytical depth and was complemented by iterative reflection and discussion among the research team. The RITA method was conducted in five phases, as described by Neal et al. (2015). The first author (MLM) reviewed audio recordings repeatedly to identify key research foci (phase 1) and gain an overall understanding. During the next phase (phase 2), the codebook was developed and refined through iterative discussions among three researchers (MLM, KD, and CH). Consensus was reached regarding the relevance and clarity of each theme in relation to the study’s aim. The last author (CH), who conducted the interviews and had also reviewed the audio recordings, contributed to validating the thematic structure.

In the following phase (phase 3), a form was developed to code while simultaneously listening to the audio recording of an interview, the presence and the valence (i.e., positive, negative, or neutral mentions) of themes. After that (phase 4), the codebook and coding form were tested (MLM), and after discussion in the research group, some minor adaptations were made. In the final phase of RITA (phase 5), all interviews were coded in the coding form by the first author (MLM). The content analysis continued by rereading and reflecting on the code forms and creating sub-themes, which led to constructing the overarching theme. To enhance transparency, the codebook and coding form template used in the RITA method are available as supplementary material (The codebook and coding form template). This dynamic analytical process moved back and forth between general and specific perspectives, meaning that the researchers alternated between reviewing overarching patterns across all interviews (general) and examining detailed, context-specific expressions within individual interviews (specific). This iterative approach supported both thematic saturation and nuanced interpretation of the data. Co-authors (MLM, KD, and CH) critically examined and discussed their understandings and interpretations during the interpretation process. Thematic saturation was assessed iteratively during the analysis process. As interviews were coded and reviewed, the research team monitored the emergence of new themes and the consistency of existing ones. Saturation was considered achieved when no new themes appeared in the final interviews and all identified themes were supported across multiple interviews. This assessment was discussed and confirmed collaboratively by the research team.

## Results

**Fig. 1:**
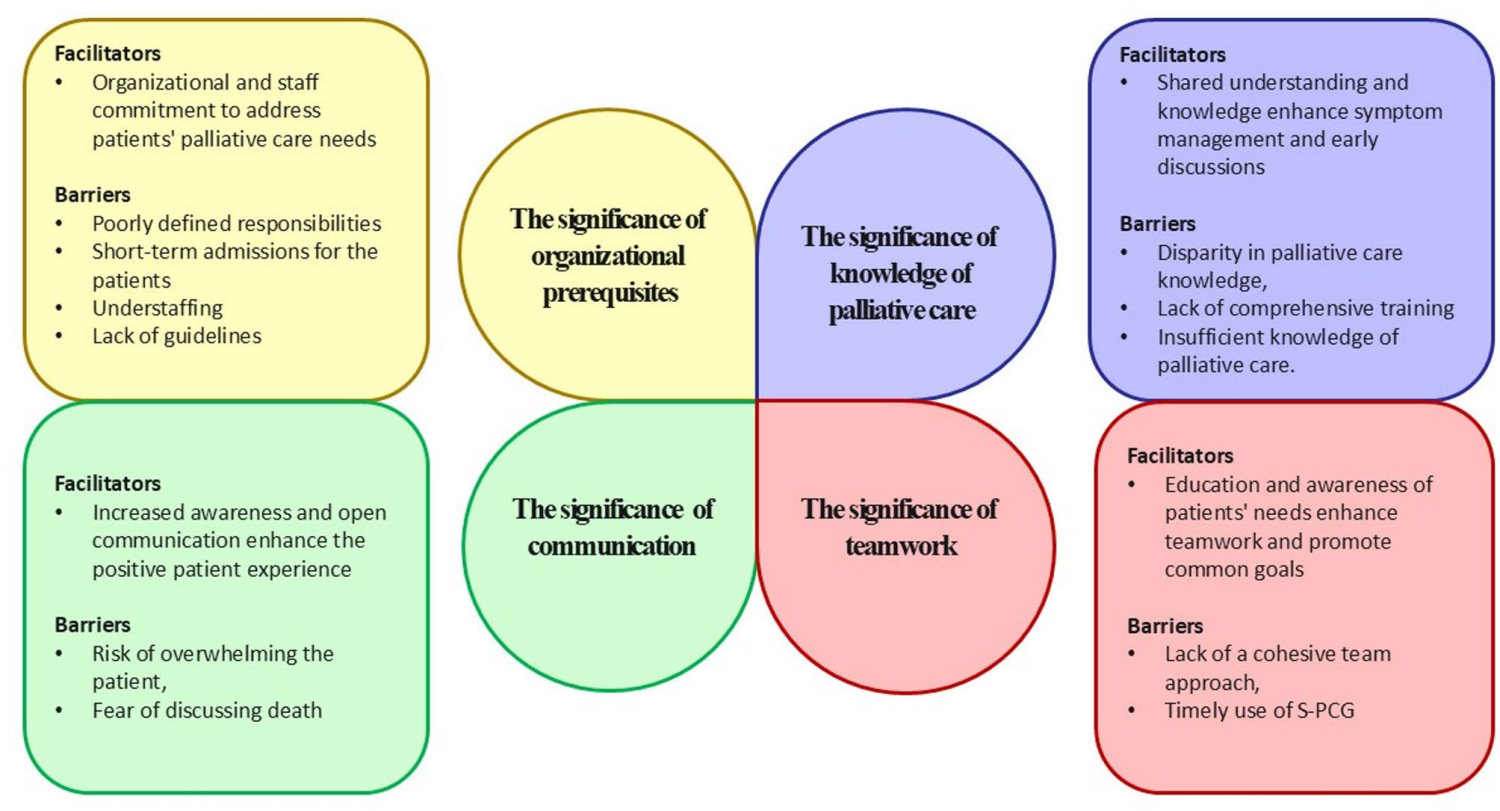
Schematic picture of the results in the four different themes

Most of the 13 participants were female (*n* = 11), and they were either nurses (*n* = 11) or physicians (*n* = 2); the age range was 29–55 years, and the professional experience ranged from 9 months to 28 years. From the participants’ perspective, integrating a palliative care approach in a geriatric setting through implementing the S-PCG is characterized by its multifaceted and complex nature, marked by facilitators and barriers. Reflective discussions within interviews have identified four interrelated themes: the significance of *(i) organizational prerequisites*, (ii) *knowledge of palliative care*, (iii) *teamwork*, and (iv) *communication*. Perspectives of these themes serve dual roles as facilitators and barriers, collectively elucidating the process of adopting a palliative approach as a transformative change in geriatric care practices (for visualization see Fig. 1).

### The significance of organizational prerequisites

From the participants’ perspective, organizational prerequisites either facilitate or hinder integrating a palliative care approach into geriatric settings.

#### Facilitators: organizational and staff commitment to address patients’ palliative care needs

One facilitator was the organizational support and emphasis on thorough preparations to ensure that all participants understood the effort required to implement a palliative care approach. Another was the participants’ willingness to adapt their practices to address patients’ palliative care needs better. Many participants also saw continuity in care as a facilitator. Having the same healthcare worker with the same patients facilitated the use of S-PCG and allowed for discussions to be distributed over several days.

*You got more out of the patients during admission*,* instead of just asking general questions like how they’re feeling. It helped us focus and catch things we might otherwise miss. G2*.

According to most participants, the structured use of S-PCG was perceived as a facilitator for the comprehensive assessment of patient needs. This structure enabled more focused and meaningful conversations during admission, which in turn supported more personalized and optimal care.

#### Barriers: poorly defined responsibilities, short-term admissions for the patients, understaffing and lack of guidelines

Many participants identified ambiguity in professional responsibilities and insufficient preparation for integrating routine care with the S-PCG as barriers. They noted insufficient guidance on when and how to use the S-PCG, leading to inconsistencies and uncertainty in its application, which complicated the seamless incorporation of the S-PCG into daily practice. Additionally, participants identified time limitations and understaffing as significant barriers to implementing the S-PCG. Some noted that short-term admissions and the exclusion of patients with cognitive impairments, further complicated the process. They experienced that these factors limited their ability to address sensitive topics comprehensively and reduced patient recruitment over time, contributing to the S-PCG being overlooked.

*It has fallen out of routine. If the patient is not cognitively intact*,* we skip it. And then it is not done for anyone. That is the problem*,* it is not part of the standard process G 3*.

They highlighted the need for clear guidelines and comprehensive training to ensure that all staff are prepared to apply the palliative care approach within the S-PCG.

### The significance of knowledge of palliative care

Knowledge and competence in palliative care is crucial for implementing the S-PCG in geriatric settings. Participants identified facilitators and barriers affecting the dissemination and application of this knowledge.

#### Facilitators: shared understanding and knowledge enhance symptom management and early discussions

Most participants identified that healthcare workers’ shared understanding of the effort required to implement palliative care created a strong foundation for the implementation process of S-PCG. Another facilitator was that adequate knowledge of symptom management, especially pain management, was crucial. Most participants noted that structured questions from the S-PCG facilitated early detection and optimal symptom management, offering a comprehensive understanding of patient’s health conditions for better overall care.

*You start thinking about different aspects of the patient*,* like symptom relief. You can be a bit ahead*,* asking questions you normally would not during admission. It gives you a different perspective.”. G 1*.

For successful implementation, participants emphasized the importance of continuous education, regular workshops, and team meetings to foster open communication and mutual respect. Some said these initiatives were vital for maintaining knowledge and ensuring a consistent palliative care approach. From the participants’ perspectives, the educational component before the implementation of S-PCG provided a robust foundation, including tips on symptom assessment and communication. This significantly transformed their understanding of palliative care to include chronic conditions and existential issues rather than solely focusing on end-of-life care.

#### Barriers: disparity in palliative care knowledge, lack of comprehensive training and varying levels of preparedness

One barrier identified by some participants was the disparity in palliative care knowledge among colleagues, which created significant challenges. Participants explained that this discrepancy stemmed from their formal education, where palliative care received little emphasis for nurses and physicians. Some acknowledged their lack of knowledge and experience in end-of-life care and discussing patient preferences and priorities, which hindered their ability to adopt a palliative care approach and implement S-PCG effectively. Furthermore, some felt inadequately prepared before the educational component, limiting their ability to fully benefit from the training on S-PCG.

*We needed more guidance*,* especially on how to handle the deeper conversations. You can not open up a big conversation with a patient and then not know how to follow through. G 4*.

All participants emphasized the need for a shared knowledge base and ongoing training to ensure colleagues are equipped to deliver high-quality palliative care. Participants mentioned that these knowledge and preparedness gaps created inconsistencies in care delivery and communication.

### The significance of teamwork

Participants identified various facilitators and barriers that significantly influenced the effectiveness of teamwork in applying a palliative care approach.

#### Facilitators: education and awareness of patients’ needs enhance teamwork and promote common goals

Most participants found the educational component to be a significant facilitator, receiving a boost in confidence and awareness that led to better teamwork, improved information sharing, and enhanced communication, resulting in a more holistic understanding of patients’ needs, wishes, and priorities. Some noted that implementing the S-PCG had heightened their awareness of the importance of examining patients’ priorities, needs, and wishes, as well as utilizing symptom assessment scales. This fosters a more person-centred approach and enhances communication and coordination among healthcare professionals.

*We all had the same goal*,* and that made it easier to talk about things like ageing and end-of-life care. It started real team discussions. G 3*.

Participants described how the S-PCG supports comprehensive evaluations, enabling earlier recognition of patient needs and more timely, tailored support. They also observed that during the implementation of S-PCG, all team members shared the same goal, which initiated team discussions and facilitated conversations with patients about the ageing process and end-of-life care. They also experienced that continuity in care was seen as a facilitator that improved teamwork.

#### Barriers: lack of a cohesive team approach and timely use of S-PCG

A barrier was that most participants initially believed they were engaged in optimal teamwork. However, some soon realized that collaboration needed improvement when working with S-PCG. This dependency led to confusion and inefficiency when team members did not share the same understanding, resulting in fragmented care. Most also noted divergent views on managing duties, particularly between physicians and nurses, which further impeded teamwork. Additionally, disagreements regarding the timing and use of S-PCG during short inpatient care complicated collaboration. According to most participants, the unclear division of responsibilities posed significant challenges, creating inefficiencies in the care process.

*It was unclear who was responsible*,* who should follow up*,* where to document*,* and how to make sure it was done. That made it easy for things to fall through the cracks. G 5*.

Some participants noted differing expectations among team members, with initial scepticism about the complexity of S-PCG being a significant barrier. They pointed out that this scepticism, along with varying levels of preparedness and awareness of palliative care, led to confusion and inefficiency, highlighting the need for a shared knowledge base to ensure cohesive teamwork. Participants described working in parallel within distinct roles, with limited opportunities for shared decision-making or joint care planning. This was evident in the fragmented documentation and unclear responsibilities described by several participants. The implementation of the S-PCG challenged these routines by requiring more integrated collaboration.

### The significance of communication

The significance of communication in providing palliative care was an essential element in implementing the S-PCG in geriatric settings. Participants identified both facilitators and barriers that influenced their effectiveness.

#### Facilitators: increased awareness and open communication enhance positive patient experience

Some participants identified increased awareness of discussing patients’ priorities, needs, and wishes as a significant facilitator. Additionally, some reported that the S-PCG facilitated and promoted early discussions about illness and its trajectory, preparing patients for future changes in their health status and empowering them with knowledge. According to some participants, the educational component improved communication of sensitive topics, such as patient priorities and impending death. Participants found that the S-PCG promoted open communication, encouraging them to ask questions they would not usually ask. This approach helped them identify symptoms earlier and facilitate more open discussions about illnesses with patients.

*It gave patients a chance to talk freely*,* not just answer boring questions like where they live. We got to know what they really feel and think. G 1*.

Some reported that this openness reduced patients’ anxiety, leading to positive experiences with S-PCG, where patients felt seen and heard. Additionally, most participants found that implementing S-PCG enhanced patient care by enabling early detection of needs, which was crucial given the short-term admissions.

#### Barriers: Risk of overwhelming the patient and fear of discussing death 

Most participants considered communication within the team crucial for addressing the responsibilities of different professionals within the S-PCG and avoiding overwhelming patients with repetitive inquiries. Additionally, some had reservations about discussing death in a geriatric setting, fearing adverse reactions from patients when addressing sensitive topics. They explained that this fear often leads to the avoidance of these conversations, hindering the application of a palliative care approach.

*I have no problem discussing symptoms and relief*,* but when it comes to talking about death*,* I worry about how patients will react. G 5*.

Here, the participants again highlighted the need for clear guidelines and thorough training to ensure that all staff are prepared to apply the palliative care approach within S-PCG.

## Discussion

The results of this study provide valuable insights into the facilitators, barriers, and sustainability issues associated with integrating a palliative care approach in geriatric settings using the S-PCG. Key factors for successful implementation and sustainability include ongoing organizational support, continuous education, a shared understanding of palliative care, teamwork, and open communication. Several barriers hindered the integration and sustainability of a palliative care approach in geriatric settings, including unclear division of responsibilities, lack of cohesive teamwork, insufficient training, time constraints, and reluctance to discuss end-of-life issues. These challenges were particularly evident during short-term admissions and in settings with limited continuity of care.

Participants described the implementation of the S-PCG as a complex and multifaceted process. This complexity reflects the guide’s comprehensive scope, which includes identifying palliative care needs, assessing symptoms, supporting decision-making, and tailoring interventions [28]. Several barriers to implementation were identified, consistent with previously reported challenges such as poor interprofessional communication [33]. Our findings further highlight persistent issues, such as unclear professional roles, fragmented teamwork, and disparities in palliative care knowledge.

Participants in this study emphasized that successful implementation of the S-PCG required strong organizational support, clearly defined roles, and continuity of care. These findings align with established principles in implementation science, emphasizing that leadership engagement, system-level planning, and structured decision-making are essential for embedding palliative care into routine practice [34]. They argue that without adequate organizational infrastructure, even well-designed interventions may struggle to achieve sustainable impact. Similarly, Hasselaar et al.[35] argue that integration must be supported not only by clinical tools but also by a strong organizational culture and policy framework. In contrast, even minimal organizational changes—such as the introduction of simple decision checklists—can lead to short-term improvements in care quality. However, their long-term sustainability remains uncertain without broader organizational support [34]. Our findings align with the growing consensus that successful implementation of palliative care approaches requires more than individual effort or isolated tools for identifying and addressing palliative care needs.

Disparities in palliative care knowledge and insufficient previous training were identified as key barriers to implementing the S-PCG. Participants emphasized the need for ongoing education and a shared understanding of palliative care principles. These findings align with previous research showing that palliative care is often underrepresented in medical and nursing education, contributing to variability in practice [35, 36]. In our study, knowledge of symptom management, particularly pain relief, was considered essential for early detection and intervention. Fallon et al. [37] demonstrated that structured pain assessment tools, combined with staff training, significantly improved timely pain relief in patients with cancer. Similarly, Basch et al.[38] found that routine symptom monitoring using patient-reported outcomes led to earlier interventions aimed at symptom relief and improved symptom control. These findings reinforce that healthcare professionals can intervene more effectively with proper knowledge and tools. Supporting this Schelin et al. [30] showed that integrating palliative care into geriatrics improved patient satisfaction, likely due to more timely and responsive symptom management. However, this was the parent trial of the present study, and several limitations were noted, including the cohort design and the overlap with the COVID-19 pandemic, which affected both implementation and follow-up. Participants in the current study also reflected on the discontinuation of the intervention after the trial period, highlighting challenges in sustaining palliative care integration.

In this study, teamwork was one of several key factors identified for the successful implementation of the S-PCG. Participants noted that the guide enhanced communication and coordination among healthcare professionals. Although many initially believed they were practicing effective teamwork, discussions revealed confusion between multiprofessional and interprofessional collaboration. As Körner [39] describes multi-professional teams working in parallel with distinct roles, while interprofessional teams engage in shared decision-making and joint care planning. This distinction became particularly relevant in our study, as the S-PCG required shared documentation and collaborative care planning, elements that challenged existing routines and workflows. Participants described their teams as primarily multiprofessional, with limited shared decision-making and fragmented documentation practices. These reflections highlighted a need to shift toward a more interprofessional model, characterized by closer collaboration and frequent communication to align treatment goals. This is supported by Kesonen et al. [40], who found that interprofessional teamwork facilitates high-quality palliative care. Similarly, Klarare et al. [41] demonstrated that team type and maturity significantly influence the effectiveness of specialist palliative home care. These findings reinforce the notion that well-designed tools like the S-PCG may fall short of their full potential without a cohesive team approach.

Participants reported that the guide facilitated early and open discussions about illness trajectories, patient priorities, and end-of-life preferences, enhancing positive patient experiences. This supports the quantitative findings from Schelin et al.[30] on patients’ experiences of the S-PCG in geriatric settings. Effective communication is central to person-centred palliative care and was highlighted by participants as both a facilitator and a challenge. Our findings align with broader evidence that emphasizes the importance of empathetic, timely, and tailored communication in improving patient satisfaction and emotional well-being. The EAPC White Paper [2] reinforces this by identifying communication and shared decision-making as key domains in an integrative approach to care for older people living with frailty. These insights support the need for structured tools and training to help healthcare professionals navigate sensitive conversations and deliver care that aligns with patients’ values and priorities.

### Strengths and limitations

A key strength of this study lies in its qualitative design, which enabled in-depth exploration of healthcare professionals’ experiences with implementing the S-PCG. The use of a consistent semi-structured interview guide and thematic analysis across all small group interviews and the individual interview ensured coherence and analytical rigour. The inclusion of both nurses and physicians provided insights from two key professional groups involved in geriatric care. However, we acknowledge that the relatively small number of physicians (*n* = 2) limits the diversity of perspectives, and the findings primarily reflect the nursing perspective. Although the small-group format (three participants per session) and one individual interview limited the interactive dynamics typical of traditional focus groups, these formats allowed for focused reflection and ensured that otherwise unavailable perspectives were included. Therefore, these are referred to as small-group interviews in the manuscript. Additionally, the inclusion of one individual interview, while necessary to capture an otherwise unavailable professional perspective, represents a methodological limitation. Individual interviews lack the interactive dynamics and collective reflection that characterize group interviews. However, the use of a consistent semi-structured guide and thematic analysis across all interviews helped mitigate this limitation, ensuring coherence in the data. The single-centre setting may limit generalizability, but the findings offer valuable insights into the implementation of palliative care tools in geriatric practice. Another limitation is that only one researcher conducted the coding of the audio recordings. Although the results were discussed within the research team and consensus was reached on theme relevance and interpretation, the use of the RITA method meant that other researchers did not directly compare coded data to the original audio material. This limits the ability to assess inter-coder reliability and may affect the transparency of the analytical process. A further strength is that one of the authors (CH) both conducted and listened to all interviews, which strengthened the interpretative process by enabling informed discussions based on shared familiarity with the data.

## Conclusions

This study highlights several key factors influencing the implementation of the Swedish Palliative Care Guide (S-PCG) in geriatric settings. Participants emphasized the importance of clear responsibilities, adequate training, and open communication to support the integration of a palliative care approach. While these aspects were considered in the initial development of the intervention, our findings suggest that their operationalization during implementation was inconsistent. Based on these insights, we propose several practical steps to address persistent barriers. First, responsibilities for using and following up on the guide should be clearly defined across professional roles to ensure accountability. Second, integrating the S-PCG into existing digital documentation systems may reduce fragmentation and improve usability. Third, targeted training should be provided to support staff in using the guide confidently, particularly in initiating sensitive conversations about end-of-life care. Fourth, implementation strategies should be tailored to local conditions, such as short-term admissions and high staff turnover, to ensure sustainability. These recommendations are grounded in the barriers and facilitators identified in our study and may inform future implementation strategies. For research, future studies should explore how implementation models can be adapted to different care contexts and evaluate long-term sustainability. Investigating how interprofessional teams can be supported to move from parallel work toward shared decision-making may further enhance the effectiveness of palliative care tools.

## Supplementary Information

Supplementary Material 1.

Supplementary Material 2. Interview guide

## Data Availability

The data generated and analyzed during the current study are not available for public use due to confidentiality but are available from the corresponding author upon reasonable request.

## Abbreviations

S-PCG: The Swedish Palliative Care Guide
