# Supplementary material for: Healthcare professionals’ perspectives on implementing the Swedish palliative care guide in geriatrics – a qualitative study using small-group and individual interviews
Source: BMC Geriatr. 2025 Nov 4;25:839. doi: 10.1186/s12877-025-06516-1 (PMC12584315; doi:10.1186/s12877-025-06516-1)
Supplement: Supplementary file 1 — Supplementary Material 1. [file 12877_2025_6516_MOESM1_ESM.docx]

# Code book

**Organizational prerequisites**

Factors related to institutional support, staffing, and structural conditions that affect implementation.

Facilitators: Organizational commitment, continuity in care, and structured tools.

Barriers: Unclear responsibilities, short-term admissions, understaffing, and lack of guidelines.

**Knowledge of palliative care**

Healthcare professionals' understanding and competence in palliative care principles and practices.

Facilitators: Shared understanding, adequate symptom management knowledge, and educational initiatives.

Barriers: Disparities in knowledge, insufficient training, and lack of preparedness.

**Teamwork**

The dynamics of collaboration and shared responsibilities among interprofessional team members.

Facilitators: Shared goals, improved coordination, and continuity in care.

Barriers: Fragmented collaboration, unclear roles, and differing expectations.

**Communication**

The quality and openness of interactions with patients and within the care team regarding palliative care topics.

Facilitators: Early discussions, open dialogue, and improved patient experience.

Barriers: Fear of discussing death, risk of overwhelming patients, and lack of clear communication routines.

# Coding form template

| Interview ID | Theme | Presence (Yes/No) | Valence (Positive/  Negative/  Neutral) | Text |
| --- | --- | --- | --- | --- |
|  |  |  |  |  |
|  |  |  |  |  |
|  |  |  |  |  |
