# Supplementary material for: Healthcare professionals’ perspectives on implementing the Swedish palliative care guide in geriatrics – a qualitative study using small-group and individual interviews
Source: BMC Geriatr. 2025 Nov 4;25:839. doi: 10.1186/s12877-025-06516-1 (PMC12584315; doi:10.1186/s12877-025-06516-1)
Supplement: Supplementary file 2 — Supplementary Material 2. Interview guide [file 12877_2025_6516_MOESM2_ESM.docx]

**Interview Guide for Individual and Focus Group Interviews with Staff at Geriatrics, Stockholms Sjukhem, in Connection with the Implementation of the Swedish Palliative Care Guide Part 2**

1. How have you experienced the education and implementation of the S-PCG?
2. What were your biggest concerns before the implementation?
3. What were the greatest difficulties during the implementation?
   1. Was the pre-implementation training sufficient?
4. How have you experienced the use of S-PCG Part 2?
   1. Advantages for patients and health care professionals (HCP)
   2. Disadvantages for patients and HCP
5. What were the biggest challenges when using the S-PCG?
   1. The time it takes to use it
   2. Something else?
   3. Please give examples
6. Did you experience improved care for the patients?
   1. Please give examples
7. Were any of the questions in the S-PCG particularly easy or difficult?
8. In what way has the S-PCG affected your work with the patients?
9. Di you have any suggestions for improvement in the implementation/use of the S-PCG?
10. Do you wish that the ward will continue to use S-PCG after the end of the study?
    1. If yes, please elaborate
    2. If no, please elaborate
11. What knowledge gaps in palliative care do you perceive?
12. What educational needs in palliative care do you have?
13. How do you use the S-PCG in practice (admission, during the week…)?
    1. What is easy?
    2. What is difficult?
14. Other comments
